# Relationship between physical activity and musculoskeletal disorders among low income housewives in Kuala Lumpur: A cross sectional study

**DOI:** 10.1371/journal.pone.0274305

**Published:** 2022-10-06

**Authors:** N. Z. M. Saat, Siti Aishah Hanawi, Nor M. F. Farah, Hazlenah Hanafiah, Anis Afiqah Zuha

**Affiliations:** 1 Biomedical Science Programme, Centre of Community Health (ReaCH), Faculty of Health Sciences, Universiti Kebangsaan Malaysia, Kuala Lumpur, Malaysia; 2 SOFTAM, Faculty of Information Science and Technology, Universiti Kebangsaan Malaysia, Bangi, Selangor, Malaysia; 3 Occupational Therapy Programme, Centre of Community Health (ReaCH), Faculty of Health Sciences, Universiti Kebangsaan Malaysia, Kuala Lumpur, Malaysia; 4 Faculty of Computer and Mathematical Science, Universiti Teknologi MARA Sabah Branch, Sabah, Malaysia; Universitat de Valencia, SPAIN

## Abstract

Housewives play a crucial role in their families’ and communities’ quality of life. However, musculoskeletal disorders are common among housewives due to housework and lack of physical activity. These musculoskeletal problems have been associated to leisure physical activity. As a result, the goal of this study was to determine the incidence of musculoskeletal problems and their association to physical activity among low-income women in Kuala Lumpur. A cross-sectional research was done among 82 housewives in Kuala Lumpur’s low-income housing area. Housewives were asked to fill out a sociodemographic questionnaire, a short version of the International Physical Activity Questionnaire (IPAQ), the Nordic Musculoskeletal Questionnaire (NMQ), and body mass index were measured by researcher. The total prevalence of musculoskeletal problems was found to be 89.0%, with the lowest frequency in the lower back (35%), followed by the knee (31%), ankle or foot (31%), and elbow (1% %). The subjects spent an average of 117.6 + 474.8 minutes per week on MVPA and 524 + 810.3 MET minutes per week on physical activity. A total of 88% of the participants had a low level of physical activity, 8.5 percent had a moderate level of activity, and 3.7% had a high level of activity. In a Chi-square test, there was no significant association between musculoskeletal problems and physical activity. The primary sociodemographic factors impacting musculoskeletal diseases were body mass index and household income, while the primary variables influencing physical activity were household income and education level, according to a logistic regression test. In a Poisson regression test, only body mass index had a significant relationship with number of musculoskeletal disorders. In conclusion, housewives are likely to suffer from musculoskeletal problems, with a high incidence in the lower back, knees, ankles, and feet, and the majority of them engage in little physical activity. Furthermore, the high percentage of musculoskeletal problems in this study varies with the findings of other research based on the type of housework done by housewives in low-cost housing areas. Future research should look at identifying the sorts of household tasks and positions employed, as well as the amount of hours spent on housework each week.

## Introduction

Musculoskeletal disorder is defined as pain or discomfort in any of the nine body areas: the neck, shoulder, elbow, wrist/hand, upper back, lower back, knees/thigh, hip/thighs, and ankle/feet [1]. Musculoskeletal disorders (MSDs) are a leading cause in Western countries such as Brazil, Canada, and Italy, where research has previously been conducted [2, 3]. MSD are rather prevalent, with prevalence rates ranging from 11% to 60% [4]. In 2013, there were 632 million individuals suffering from back pain, 332 million suffering from neck discomfort, 251 million suffering from knee osteoarthritis, and 561 million suffering from various MSD globally [5].

Consistent physical exercise can bring several benefits, particularly for excellent and long-term health. Physical activity can reduce muscle discomfort, preserve function, and avoid cardiovascular diseases [6]. Any movement of the body induced by skeletal muscles that needs energy expenditure, such as work, play, household responsibilities, travel, and leisure activities, is considered physical activity [7] According to the WHO, adults should engage in at least 2.5 hours of moderate and 75 minutes of vigorous physical activity per week, which is comparable to 600 metabolic equivalent (MET) minutes of total physical activity [8]. According to the Malaysian Adult Nutrition Survey (MANS), around 7.1 million individuals in Malaysia are physically inactive, indicating less than 600 MET per week, contributing to 36.9% of the adult population aged 18 to 59 years [9].

MSD affects the muscles, nerves, ligaments, tendons, joints, cartilage, and lesions or pain in the spinal rope [10]. Shoulders, neck, elbows, hands/wrists, upper back, hips/thighs, lower back, knees, and ankles/feet are nine body areas at risk for MSD [11]. In Malaysia, 95% of sewing machine employees, the most of whom are female, suffer from neck or shoulder discomfort [12]. A study conducted among Malaysian dental students revealed that the majority of the discomfort was in the neck, shoulder, and lower back as a result of repetitive motions and vibrating tools [13] This reflects the discomfort symptoms that individuals have as a result of their daily occupation. Local or systemic discomfort, irritation, loss or hypersensitivity to touch, heat and pressure, loss of muscular strength, and inability to perform coordinated motions or balance response are all symptoms of MSD [14]. Housework that includes manual handling, prolonged sitting and standing, bending, and repeated motions may cause MSD to develop over time, impacting a person’s personal and social life as they experience discomfort when doing housework and engaging in a leisure activity such as gardening [15]. The development of MSD is interrelated to postural demands [16].

Housewives do a variety of housework, which contributes to increased ergonomic stress and MSD [5]. Housework may be a key risk factor leading to the development of MSD in women, as evidenced by the high incidence of MSD among housewives in Iran [17] and India [18]. Women have a greater frequency of MSD than males, according to both general and occupational research. This may be due to a lower pain threshold, as one study reported lower pain tresholds among women than among men [19]. Furthermore, research have revealed that women are more likely than males to engage in repetitive labour and carry out their tasks with an uncomfortable body position [20].

Although there have been many research in Malaysia regarding MSD, there have been few research focusing housewives as subjects. As a result, the goal of this study was to determine if there was a relationship between physical activity and MSD, BMI and socio demographic factors among housewives in low-income areas of Kuala Lumpur.

## Materials and methods

### Study and sampling participants

This study was approved by the Universiti Kebangsaan Malaysia Research Ethics Committee UKM PPI/111/8/JEP-2019-825This cross-sectional study was carried out in Kuala Lumpur’s five low-cost housing neighbourhoods. Housewives who met the inclusion criteria which is age above 21 years old, married, does not have physical disability and volunteered to participate were included in the research. The exclusion criteria were those who were disabled, pregnant, or had a history of MSD. The sampling method is purposive sampling. The sample size of the study was using formulae $n=\frac{\left(Z{)}^{2}p(1-p\right)}{\Delta^{2}}$ p [20] is the prevalence of MSD among housewives which is 53% [16], z = 1.96, Δ = 10%. $n=\frac{\left(1.96{)}^{2}0.53(1-0.53\right)}{0.10^{2}}$. Therefore 95 respondents were required for this study.

The included housewives were given a self-administered and guided questionnaire that included socio-demographic information such as age, time as a housewife, number of children, education, and household income, as well as the International Physical Activity Questionnaire (IPAQ)-short form and the Nordic Musculoskeletal Questionnaire (NMQ). The person who accepted to participate in the study completed a document verifying her informed permission.

### Instruments and procedures

Weight is measured using an analog scale with the nearest to 0.1 kg, without shoes, wore light clothing and items were removed from the pocket before being weighed. While the height is measured to the nearest 0.5 cm by using a portable height measuring device (Seca 213). Body Mass Index (BMI) is calculated as weight in kilograms divided by height in metres squared (kg m^-2^) and categorized according to cut-off points for Asians, with underweight (<18.5), normal (18.5–22.9), overweight (23.0–27.49) and obesity (≥ 27.5).

Through an interview session, the physical activity was measured using a short form of the International Physical Activity Questionnaire (IPAQ). The questionnaire has been evaluated and verified among Malaysia’s Malay community for its reliability [21] This section contains seven questions about physical activity, including vigorous-intensity, moderate-intensity, walking, and sitting, that were completed in the previous seven days. During the interview, information was gathered on the amount of time spent conducting physical activities on weekdays and weekends.The physical activity was scoring by using the formula as follows:


MET‐min/week=minofactivity/dayxdayperweekxMETlevel

Total minutes spent on vigorous activity, moderate-intensity activity, and walking over the last 7 days were multiplied by 8.0, 4.0, and 3.3 respectively to compute MET scores for each activity. The total physical activity score was calculated as the sum of all MET scores from the three sub-components. The level of physical activity is classified into three categories which are low (<600 MET-minutes / week), moderate (600–3000 MET-minutes / week) and high (≥ 3000 MET-minutes / week) according to the guidelines of IPAQ [22].

A Nordic Musculoskeletal Questionnaire (NMQ) was used to assess MSD during an interview session. The NMQ is a broad questionnaire with 40 closed-multiple choice questions that identifies nine body locations that cause MSD issues. The MSD evaluation was examined using a binary response (yes/no) in the past 7 days and 12 months [23]. The NMQ questionnaire has been test for reliability and validity by previous researcher [24].

### Data analysis

The Statistical Package for the Social Sciences was used to analyse the data (IBM SPSS Statistics Version 25.0). Chi-square was employed to analyse the relationship between groups. The Poisson regression method was used to investigate the association between the number of MSD and sociodemographic factors. The association between MVPA and BMI, age, and MSD category (yes/no) was determined using negative binomial regression.

## Results

Table 1 summarises the demographics of the 82 women who took part in this study, as well as the percentages of housewives who reported MSD ailments. The participants ranged in age from 21 to 60 years old. The women in the study were predominantly between the ages of 51 and 60 (39.0 percent) and had been housewives for more than 20 years (43.9 percent). According to this report, just 4.9 percent of women have completed a university education. Participants were mostly from low-income families, with 91.5 percent reporting monthly family incomes of less than RM3000. According to the survey, MSD were reported by 89 percent of women. Musculoskeletal disorders percentage were shown to be increasing according to age, marital duration, BMI, and the number of children.

**Table 1:** Percentages of the housewives reporting MSD according to socio-demographic factors (n = 82).

Characteristics	n (%) Reporting MSD
**Age**	
21–30	4 (5.5)
31–40	14 (19.2)
41–50	25 (34.2)
51–60	30 (41.1)
**Education level**	
Primary	9 (12.3)
Lower secondary	14 (19.2)
Upper secondary	46 (63.0)
Certificate and above	4 (5.5)
**Monthly income**	
<RM1000 (<USD 223)	12 (16.4)
RM1001-2000(USD223-USD447)	38 (52.1)
>RM2001(>USD 447)	23 (31.5)
**Marriage duration (years)**	
<5	15 (20.5)
6–10	15 (20.5)
11–15	8 (11.1)
16–20	3 (4.1)
>20	32 (43.8)
**Number of children**	
<2	12 (16.4)
3–4	36 (49.4)
>5	25 (34.2)
**BMI**	
Underweight (<18.5)	0 (0.0)
Normal (18.5–22.9)	9 (12.3)
Overweight (23.0–27.49)	20 (27.4)
Obesity (≥27.5)	44 (60.3)
**Sedentary behaviour**	
Sit <4 hours	51 (69.9)
Sit >4 hours	22 (30.1)

**Fig 1:**
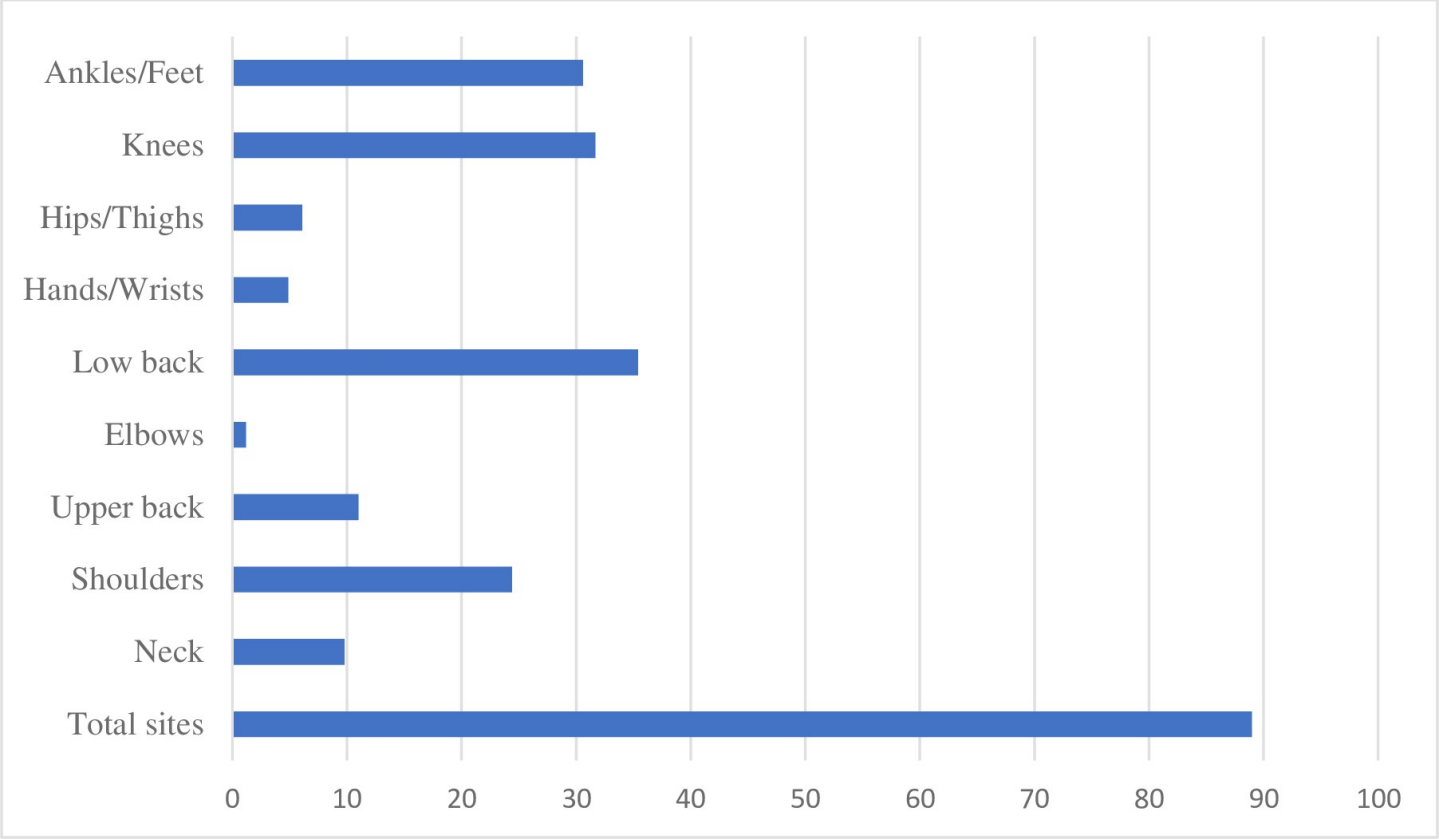
Location of MSD among housewives.

Fig 1 depicts the percentage of Kuala Lumpur housewives with MSD in nine different body locations. The most common areas for MSD were found to be the low back (35.4%), knees (31.7%), and ankle/feet (31.7%).Table 2 demonstrates that the average of respondents reported low physical activity (87.8%), moderate physical activity (8.5%), and high physical activity (3.7%).This study’s overall median of total physical activity is 244± 445MET-min/week, demonstrating that housewives do not engage in significant physical exercise. Housewives were reported to be physically inactive since they did not meet the recommended 600 MET-min/week comparable to 150 minutes of moderate-vigorous physical activity. (90± 48 MET-min/week) (MVPA). Nearly everyday, a housewife spends an average of 180±120 min/week sitting.

**Table 2:** Status of physical activity based on IPAQ (n = 82).

Parameter	n (%)	Median ± IQR
**Category of physical activity level**		
Low	72 (87.8)	-
Moderate	7 (8.5)	-
High	3 (3.7)	-
**Physical activity level**		
Total physical activity level (MET-min/week)	-	244 ± 445
MVPA (MET-min/week)	-	90 ± 48
Sitting (minutes/week)	-	180 ± 120

Table 3 further describes the relationship between physical activity levels and sociodemographic factors. The percent of respondents who are inactive are older, overweight or obese, have a lower level of education, and have lower monthly salaries. Table 4 demonstrates the connection between MSD and physical activity levels in nine body areas. The Chi-square test revealed that there was no significant association between MSD regions and physical activity level.

**Table 3:** Distribution of physical activity level with socio-demographic characteristics.

Characteristics	Physical activity level		
	Low(n = 72) n (%)	Moderate (n = 7) n (%)	High (n = 3) n (%)
**Age**			
21–30	4 (5.6)	0 (0.0)	0 (0.0)
31–40	17 (23.6)	0 (0.0)	2 (66.7)
41–50	22 (30.6)	4 (57.1)	1 (33.3)
51–60	29 (40.3)	3 (42.9)	0 (0.0)
**Education level**			
Primary	10 (13.9)	0 (0.0)	0 (0.0)
Lower secondary	11 (15.3)	3 (42.9)	0 (0.0)
Upper secondary	48 (66.7)	3 (42.9)	3 (100.0)
Certificate and above	3 (4.2)	1 (14.2)	0 (0.0)
**Monthly income**			
<RM1000(<USD 223)	12 (16.8)	2 (28.6)	0 (0.0)
RM1001-2000(USD 223-USD447)	37 (51.4)	1 (14.2)	2 (66.7)
>RM2001(>USD 447)	23 (30.0)	4 (57.2)	1 (33.3)
**Marriage duration (years)**			
<5	16 (22.2)	0 (0.0)	1 (33.3)
6–10	15 (20.8)	2 (28.6)	1 (33.3)
11–15	8 (11.1)	0 (0.0)	0 (0.0)
16–20	3 (4.2)	0 (0.0)	0 (0.0)
>20	30 (41.7)	5 (71.4)	1 (33.3)
**Number of children**			
<2	13 (18.1)	1 (14.2)	0 (0.0)
3–4	34 (47.2)	4 (57.1)	3 (100.0)
>5	25 (34.7)	2 (28.6)	0 (0.0)
**Body mass index**			
Normal	10 (13.9)	2 (28.6)	0 (0.0)
Overweight	21 (29.2)	2 (28.6)	0 (0.0)
Obesity	41 (56.9)	3 (42.9)	3 (100.0)
**Sedentary behaviour**			
Sit <4 hours	50 (68.5)	5 (83.3)	2 (66.7)
Sit >4 hours	23 (31.5)	1 (16.7)	1 (33.3)

**Table 4:** The association between MSD between 9 body parts with physical activity level.

Regions of MSD		Physical activity level			
		Low	>Moderate	χ^2^	*P value*
		n (%)	n (%)		
Neck	Yes	6 (8.3)	2 (20.0)	1.357	0.244
	No	66 (91.7)	8 (80.0)		
Shoulders	Yes	17 (23.6)	3 (30.0)	0.194	0.659
	No	55 (76.4)	7 (70.0)		
Elbows	Yes	1 (1.4)	0 (0.0)	0.141	0.708
	No	71 (98.6)	10 (100.0)		
Hands/Wrists	Yes	4 (5.6)	0 (0.0)	0.584	0.445
	No	68 (94.4)	10 (100.0)		
Upper back	Yes	9 (12.5)	0 (0.0)	1.404	0.236
	No	63 (87.5)	10 (100.0)		
Low back	Yes	28 (38.9)	1 (10.0)	3.206	0.073
	No	44 (61.1)	9 (90.0)		
Hips/Thighs	Yes	4 (5.6)	1 (10.0)	0.303	0.582
	No	68 (94.4)	9 (90.0)		
Knees	Yes	24 (33.3)	2 (20.0)	0.721	0.396
	No	48 (66.7)	8 (80.0)		
Ankles/Feet	Yes	18 (25.0)	4 (40.0)	1.006	0.316
	No	54 (75.0)	6 (60.0)		

Table 5 presents the results of the Poisson regression model, to determine the relationship between total number of MSD among respondents with age, BMI and MVPA. Based on the likelihood ratio Chi Square, overall model are significant *χ*^2^ = 8.725, p = 0.003. The number of symptoms of MSD has positive relationship with BMI with exponentiated value 1.043, this indicate the number of symptoms will be 1.043 greater with the increase in BMI. 1.043 increase in the number of symptoms with the unit increase in BMI. However number of MSD was not significantly related to MVPA and age. Meanwhile according to Table 6,using the negative binomial regression there was significant relationship between MVPA with age, BMI and NMQ(yes/no). There was an inverse connection between MVPA and BMI, showing that the majority of physically active individuals have considerably lower BMI. Furthermore, there was a substantially lower incidence of MSD among physically active participants.

**Table 5:** Poisson regression model, relationship between total area of MSD with BMI, age and MVPA.

	Coefficient ß	Standard error ß	Wald	OR	(95% CI)
Intercept	-1.056	.6467	2.669	.348	(0.098,1.235)
BMI	.042	.0138	9.281	1.043*	(1.015,1.071)
Age	.005	.0099	.005	1.005	(0.985,1.024)
MVPA	4.235E-5	.0006	.212	1.000	(0.999,1.001)

**Table 6:** The results of the negative binomial regression for MVPA (as a dependent variable) age, BMI, and MSD.

Independent Variables	β	S.E of β	Wald	OR (95% CI)	(95% CI)
(Intercept)	-1.215	1.2471	.949	.297	(0.026,3.420)
BMI	-.089	.0388	5.297	.915*	(0.848,0.987)
Age	.200	.0219	82.931	1.221*	(1.170, 1.274)
MSD(yes)	-3.146	.5064	38.610	.043*	(0.016,0.116)

## Discussion

The goal of the study was to determine association between physical activity and the occurrence of MSD. In this research, 89 percent of the women had MSD. MSD were found to be prevalent in 50 percent of the population among office workers men and women in Malaysia [25]. The prevalence was substantial, comparing the percentage of MSD in this study, was higher compared to 53 percent among women in Iran [16] and 77 percent among women in Lebanon [18]. However, when comparing the percentage of housewives with MSD in this study to a study in India, the percentage was slightly lower, compared to 100% of housewives in India having MSD, attributed to housework such as cleaning and repeated lifting of heavy loads or children weighting more than 10kg [18]. As a result, this demonstrates a high prevalence of housekeeping as an independent risk factor for musculoskeletal disorders among women.

The highest percentage of MSD was seen in the low back (35.4 percent), followed by knees (31.7 percent), ankles or feet (30.6 percent), and elbows (30.6 percent) (1.2 percent). Many research from many nations such as Malaysia, Brazil, Italy,Canada and other countries found that MSD in the lower back area are the most common [2, 3, 26]. Muscle strains, instability owing to weak postural muscles, lack of flexibility of the spinal joints, and degeneration or herniation of the spinal disc might all be contributing factors [27]. Another study discovered an association between MSD in women and the items they use to clean their houses, such as mop and brooms, rather than vacuuming, which requires them to bend and kneel [16]. Housework that requires slightly uncomfortable postures such as kneeling, kneeling, and bending, such as sweeping and cleaning the floor in tight or difficult-to-reach spots, washing clothing, and lifting weights affect the lower back area. These are all repeated actions that cause spinal movement, such as bending, twisting, lifting, and tugging.

In comparison to women who did not have musculoskeletal disorders, housewives with musculoskeletal disorders had a higher BMI. There was a significant relationship between the number of MSD and BMI in this research. This conclusion is comparable to one found in a research of military veterans, which found that the higher the BMI, the greater the pain intensity among participants [28]. In a previous study, BMI was found to be highly associated with musculoskeletal symptoms in the low back, knees, ankles, and feet [29]. Being overweight or obese puts additional strain on human muscles, increasing the risk of musculoskeletal disorders [30]. Overweight and obesity, according to another study, raised the incidence of broad musculoskeletal disorders during an 11-year period [31]. Higher BMI will increase the risk of metabolic syndrome which is obesity, hypertension, hyperglycaemia and hypertriglyceridemia [32].

The participants in this study are housewives from low-income families who live in low-income housing with a monthly income of less than RM3000(USD670). According to the 1st National Health and Nutrition Examination Survey (NHANES-1), women, the elderly, and low-income persons are more likely to have chronic musculoskeletal disorders in the National Health and Nutrition Epidemiologic Follow-up Survey (NHEFS) [33]. It’s possible that it’s because people from low-income families have a hard time getting health care [34]. Another hypothesis is that the income level in the NHANES, which was done in the United States, has a wide range of changes focusing on men and women’s income levels, but MSD did not differ among low-income housewives in this study due to the small variations in income levels. In contrast to a previous study in North India, monthly income did not appear to have an influence on MSD due to the small income variation between income levels [3, 34].

Physically active housewives were found to be 3.7 percent of people in this research. The majority of the participants who took part in the survey were inactive. The low level of physical activity indicated in this study is greater than Laos (8.9%), China (9.1%), and the Philippines (6.4%), but lower than Vietnam (93.5%) [35]. In contrast, prior Malaysian surveys have found that the majority of respondents, regardless of age or occupation, are physically active (66.5%) [36]. In this study, housewives spent an average of 244 ± 445MET-min/week on physical activity. This implies that the majority of the participants did not meet the WHO’s recommended minimum level of total physical activity of 600 MET-min/week [37]. According to prior study, individuals with more spare time, such as students and housewives, prefer not to engage in physical activities with that time [38]. This can be explained one study has reported that women seemed to engage in mild to moderate intensity activities, while men participated in activities of greater intensity [39]. This is because housekeeping, such as cleaning, cooking, and babysitting, requires a low to moderate degree of effort. Being physical active is important to reduce risk of cardiovascular disease [40, 41].

Physical activity levels were shown to decline with lower income in general, with low physical activity levels being more frequent among housewives with monthly incomes less than RM2000. According to a study conducted in Korea, there is an association between family income and physical activity among Korean men and women. Due to various social and environmental barriers such as poor communities, insufficient parks and recreational facilities, a lack of free time, and a lack of exposure to social support related to exercise, people who live in low-income households have a greater difficulty participating in physical activity than people who live in higher-income households [42]. According to a research conducted in the United States, those with an annual household income of $75,000 or more are more likely to engage in 4.6 minutes of moderate-vigorous physical activity (MVPA) each day than people with an annual household income of less than $20,000 [43].

In this study, there was a substantial association between physical activity as evaluated by MVPA and MSD. This finding is consistent with another research conducted among Norwegian navy personnel, which found that physical activity was inversely related to MSD [44] However, research involving physical activity and musculoskeletal disorders in nurses, office employees, and post office clerks revealed that an increase in physical activity is directly related to the number of body parts implicated in musculoskeletal disorders [45, 46]. Good lower-extremity muscular strength may help to avoid musculoskeletal disorders in the hips/thigh, knees, and ankle/foot to some extent, but attaining such strength necessitates intense physical activity, which raises the risk of acute and overuse injuries as well as musculoskeletal disorders [47]. Another study in India, discovered that engaging in physical exercise lowered the prevalence and severity of musculoskeletal diseases [48]. Evidence on the role of physical activity in musculoskeletal disorders, according to prior study is less consistent with this study [48].

Furthermore, the results of this research suggest that there is an association between monthly income and sedentary behaviour. A study conducted in low-middle-income nations found an association between socioeconomic level and sedentary behaviour [49]. Furthermore, a research in Thailand revealed that when wealth rises, so does sedentary behaviour, as seen by increased use of smartphones, laptops, computers, and video games [50]. Similar to a research in the United States that found that those with an annual household income of $75,001 or more spent 11.8 minutes more undertaking sedentary behaviour than people with an annual household income of less than $20,000 [43].

This study has a number of limitations. Since several respondents were preoccupied with housework and caring for their children, the overall sample size desired in this study was influenced. Second, some of the respondents were not at home when the data was collected, making it impossible to reach them due to a lack of information such as a phone number. As a consequence, there is a need to raise awareness about the risks of musculoskeletal disorder among residents of low-income housing areas.

## Conclusion

In conclusion, housewives are more prone to acquire musculoskeletal disorders, with a high prevalence in the lower back, knees, ankles, and feet, which may be connected to improper body posture when conducting housework. The vast majority of them are physically sedentary. As a result, promoting a healthy lifestyle while preventing musculoskeletal disorders should be a consideration.

## Supporting information

S1 Data (SAV)
